# Exploration of the Conformational Dynamics of Major Histocompatibility Complex Molecules

**DOI:** 10.3389/fimmu.2017.00632

**Published:** 2017-05-29

**Authors:** Saeko Yanaka, Kenji Sugase

**Affiliations:** ^1^Department of Life and Coordination-Complex Molecular Science, Biomolecular Functions, Institute for Molecular Science, National Institutes of Natural Sciences, Okazaki, Japan; ^2^Department of Molecular Engineering, Graduate School of Engineering, Kyoto University, Kyoto, Japan

**Keywords:** major histocompatibility complex, stability, nuclear magnetic resonance, relaxation dispersion, conformational dynamics, transient induced-fit model

## Abstract

Major histocompatibility complex (MHC) molecules are loaded with a wide variety of self- and non-self-peptides in their binding grooves and present these to T cell receptors (TCRs) in order to activate the adaptive immune system. A large number of crystal structures of different MHC alleles with different bound peptides have been determined, and they have been found to be quite similar to one another regardless of the bound peptide sequence. The structures do not change markedly even when forming complexes with TCRs. Nonetheless, the degree of TCR activation does differ markedly depending on the peptide presented by the MHC. Recent structural studies in solution rather than as crystals have suggested that the conformational dynamics of MHC molecules may be responsible for the MHC stability differences. Furthermore, it was shown that the conformational dynamics of MHC molecules is important for peptide loading and presentation to TCR. Here, we describe the static and dynamic structures of MHC molecules and appropriate methods to analyze them. We focus particularly on nuclear magnetic resonance (NMR), one of the most powerful tools to study dynamic properties of proteins. The number of such studies in the literature is limited, but in this review, we show that NMR is valuable for elucidating the structural dynamics of MHC molecules.

## Introduction

A wide variety of self- and non-self-peptides is bound by major histocompatibility complex (MHC) molecules and presented to T cells, some of which may carry specific T cell receptors (TCRs) that when ligated can activate these essential adaptive immune system cells. The degree of the resulting activity differs markedly depending on the manner in which the peptides are presented on MHC molecules. The mechanism of molecular recognition of the MHC–peptide complex by the TCR has been widely investigated, in particular from the viewpoint of structural and biophysical properties such as stability and peptide-binding kinetics. Since the early 1990s, many crystal structures of MHC–peptide complexes have been reported, even including some tertiary peptide–MHC–TCR complexes. These crystal structures contributed greatly to our understanding of the mechanisms of molecular recognition at the atomic level (1). These crystal structures were all found to be quite similar to one another; thus it is difficult to explain differences in T cell activation solely on the basis of the crystal structures. Thermodynamic characteristics of the interaction between MHC and TCR have also been probed using ITC (2). It was found that the binding affinity of MHC–peptide complexes to TCRs does not vary appreciably between any pair of MHCs and TCRs, even if they show different TCR activity and specificity. Meanwhile, the thermodynamic parameters such as Δ*C*_p_ suggested that some conformational change must occur upon binding, which could be detectable by calorimetry but cannot be discerned by examining crystal structures. Because it is generally appreciated that each crystal structure is only a snapshot of one of many possible states in solution, the crystal structures of MHC molecules, which are similar to each other, would correspond to the most stable state in solution. This suggests that conformational dynamics in solution could contribute to the differences in the MHC stability, resulting in activatory capacity despite similar binding affinities of different MHC–peptide complexes with TCRs. To explain the inconsistency between affinity and activity, one may also raise concerns about the avidity effect caused by clustering of MHC molecules on the cell surface. It has been shown that affinity on the two-dimensional surface membrane varies more than when it is determined by the regular analysis performed in solution (3). This suggests that clustering of the MHC molecules on the cell surface is important for their interaction with TCRs. For effective clustering of active MHC molecules, stable presentation of the peptides and maintenance of the integrity of active MHC structures on the cell surface are important. Several biophysical and cell biological studies have addressed the importance of the stability of the peptide-bound MHC molecules on their stimulatory activity (4, 5). Recently, several groups including our own reported an MHC stabilization mechanism discovered by focusing on conformational dynamics in solution (6, 7). In this review, we first describe the similarity of the static crystal structures and differences in activity and thermostability of MHC molecules, and then introduce recent studies of MHC dynamics that explain the stability of the complexes influencing peptide presentation. We also describe *R*_2_ relaxation dispersion nuclear magnetic resonance (NMR) spectroscopy, which we first applied to MHC molecules, documenting it as a useful method for elucidating the conformational dynamics of MHC molecules in solution.

## Structural Similarity of MHC Molecules in Complexes with Different Peptides: Differences in Thermal Stability

The stability of peptide–MHC complexes is essential for the efficient activation of T cells. Some MHC alleles, such as HLA-A2, can exist even in a peptide-free form (8), which enables the precise characterization of the peptide–MHC complex formation process and of the influence of the peptide on peptide–MHC stability. However, the stability of most human MHC molecules is dependent on peptide binding; thus, the peptide-free form of MHC molecules aggregates quickly, hampering a comparative analysis of the peptide-free and bound forms. It is important to understand the mechanism of MHC stabilization by peptides, in particular because the difference in stability is relevant to some diseases, as outlined below. The thermodynamic profile of MHC molecules has been characterized by several groups (4, 5, 9). Ziegler et al. used circular dichroism and differential scanning calorimetry to examine differences in thermostability in MHC alleles with a single amino acid difference but still presenting the same peptides (5). They found that the MHC allele B*27:05, which is strongly associated with spondyloarthropathy (SA), had a different thermostability profile compared to non-SA-associated alleles. Motozono et al. showed that the stable presentation of peptides originating from the HIV envelope is associated with the long-term non-progression of autoimmune syndrome disease (4). In that study, the impact of thermal stability on activity decay was measured in a quantitative manner using a cell-based assay. It was found that even only a single amino acid substitution in the HIV peptide leads to a large difference in the thermal stability.

**Figure 1:**
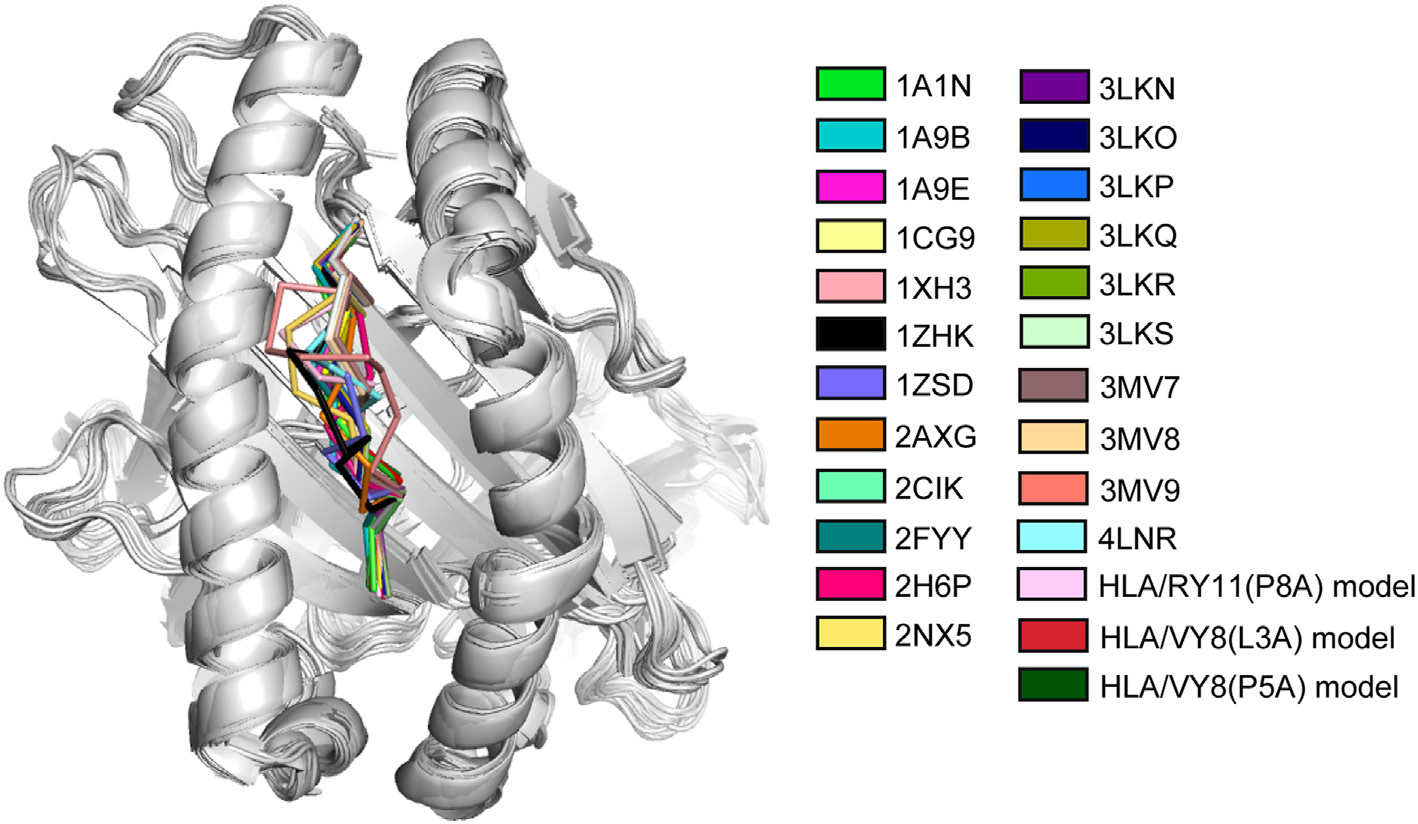
**Superimposition of crystal and modeled structures of major histocompatibility complex (MHC) molecules**. The peptide of each MHC molecule is colored as indicated with the PDB accession code. The last three modeled structures were constructed in the study of Ref. (7). The figure is reprinted from Ref. (7).

The MHC class I molecule–peptide complex consists of three components: the polymorphic heavy chain, β2 microglobulin M (β2M), and the short bound peptide. The heavy chain is the major component that mainly determines the stability of the MHC and possesses the region where the peptide binds, called the peptide-binding groove. Comparing crystal structures of MHC molecules with different bound peptides reveals that they are quite similar to one another (Figure 1), although they exhibit different thermostability. The root-mean square deviations are less than 2 Å between these structures. The structural difference is mainly observed in the peptides presented on the MHC molecules. The heavy chain possesses a well-conserved backbone structure, but the side chains are slightly different. Nonetheless, the small structural differences found in the crystal structures cannot explain the large thermostability difference. Therefore, several studies have emphasized the importance of examining the structural states in solution, including conformation dynamics.

## Spectroscopic and Mass Spectroscopy (MS) Studies on the Stability of MHC Molecules

Stabilization of MHC molecules has been examined by monitoring their disintegration or association with peptide (6, 10, 11). Ziegler et al. used fluorescence microscopy to show that MHC disintegration occurs by the dissociation of either the peptide or β2M (10). This indicates that the binding of both peptide and β2M is necessary for the stability of the complex and presentation of the peptide. Using a combination of MS and the hydrogen/deuterium (H/D) exchange reaction, Hawse et al. found that the helical regions, constituting the peptide-binding groove, show faster H/D exchange (6). This suggests that some conformational fluctuation persists in the peptide-binding groove. These investigators also suggested the possibility that crystal structures can only explain the energetically global minimum structure. An infrared spectrometry study revealed some structural differences in the heavy chains of MHC molecules in solution, although their crystal structures were very similar to one another (12). These studies suggest a certain degree of dynamic binding and unbinding events of either the peptide or β2M, and the authors speculated that the difference in the solution structure of MHCs is a determinant of their molecular stability. Therefore, to understand MHC stabilization mechanisms, it is necessary to analyze conformational dynamics of MHC molecules in solution at atomic resolution. In this regard, NMR is the most suitable method to analyze conformational dynamics in solution. In the next section, we describe technical aspects of NMR and its application to studying MHC molecules.

## NMR Studies on the Conformational Dynamics of MHC Molecules

**Figure 2:**
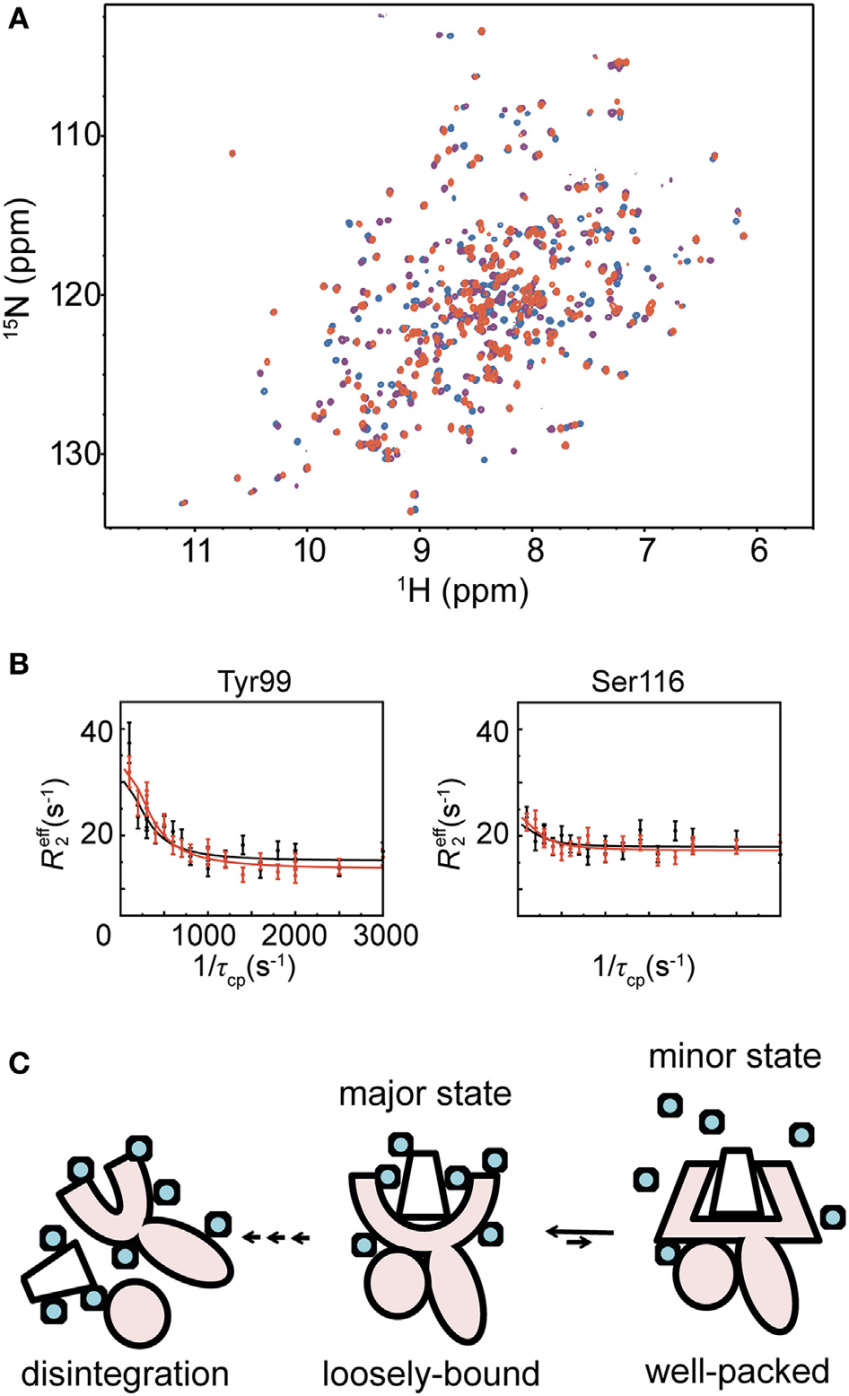
**Analysis of major histocompatibility complex (MHC) molecules in solution using nuclear magnetic resonance, and their stabilization**. **(A)** ^1^H-^15^N TROSY HSQC spectra of an MHC molecule binding different peptides are shown in red (high-stable MHC), purple (middle-stable MHC), or blue (low-stable MHC), respectively. **(B)** Representative *R*_2_ relaxation dispersion profiles of the high-stable MHC. The *R*_2_ relaxation dispersion data were collected at ^1^H frequencies of 600 MHz (black) and 750 MHz (red). **(C)** Schematic illustration of MHC fluctuation. The MHC heavy chain is shown in pink, and the peptide and β2M are represented as a trapezoid and a circle, respectively. Water molecules are shown as light blue circles. The figure is reprinted from Ref. (7).

Nuclear magnetic resonance is a powerful method to examine the conformation of proteins in solution. However, the sensitivity of NMR signals is dependent on the molecular weight of the protein to be analyzed, becoming lower with increasing molecular weight. This limits the analysis of high molecular weight proteins. Especially for quantitative analysis of conformational dynamics, a realistic molecular weight limit is ~30 kDa, although several groups have attempted to study proteins over 100 kDa (13). Nonetheless, studying MHC molecules of approximately 46 kDa is difficult using NMR. Most published NMR studies on MHC molecules were conducted during the last decade. In 2007 and in 2014, Varani et al. and Hawse et al. characterized the interaction between H-2L^d^ MHC and TCR using NMR and identified those TCR interaction sites, which had partial chemical shift assignments of the heavy chain (14, 15). In 2013, Kurimoto et al. reported NMR observations of β2M (12 kDa) and MHC (16, 17). Using amino acid selective labeling of the heavy chain, they showed that the peptide-free form of the MHC is partially unstructured. Recently, we reported fluctuation profiles of the heavy chain of a hyperstable MHC molecule that can endure extended NMR measurement times (>1 week). In that study, perdeuteration of the protein and a modified refolding protocol resulted in a great improvement of NMR signals of the MHC heavy chain (7) (Figure 2A). Inspecting the Biological Magnetic Resonance Bank, we recently noted that Ballaschk et al. had also succeeded in detecting chemical shift assignments of MHC molecules.

The improving quality of NMR data in terms of sensitivity and signal dispersion now enables a study of the detailed dynamics of MHC molecules. We recently elucidated the conformational dynamics of MHC molecules in a site-specific manner using *R*_2_ relaxation dispersion NMR spectroscopy. Here, we briefly describe this NMR approach. Many NMR methods have been established for analyzing the dynamics of proteins on different timescales, but *R*_2_ relaxation dispersion is one of the most powerful means to analyze conformational dynamics that are relevant to biological functions. This approach reveals kinetic and thermodynamics parameters of fluctuation between different conformations that interconvert in solution on sub-microsecond to millisecond timescales. These are the timescales of many important biochemical reactions, such as enzyme catalysis, protein–ligand interactions, and protein folding. When a protein interconverts between two conformational states in solution on the sub-microsecond to millisecond timescales, the effective transverse relaxation rate ^eff^*R*_2_, which determines the line width of an observed NMR signal, changes according to the ratio of the exchange rate *k*_ex_ to the chemical shift difference between the two states Δω, which represents the amplitude of the conformational change between the two states. *R*_2_ relaxation dispersion can probe the conformational change even in cases where one of the populations is skewed (~1%), which is unobservable to most biophysical methods. In the *R*_2_ relaxation dispersion measurement, the Carr–Purcell–Meiboom–Gill (CPMG) pulse, which is composed of consecutive 180° pulses with a certain interval τ_CP_, is applied to a most commonly ^15^N-labeled protein. Other types of isotope-labeled proteins can also be used to analyze, for example, relaxation dispersions of ^13^CH_3_, ^13^CO, ^13^Cα, ^13^Cβ, and other isotopes (18). The effective relaxation rate ^eff^*R*_2_ varies depending on the CPMG interval τ_CP_. By fitting the obtained ^eff^*R*_2_ data at different τ_CP_ to a theoretical equation, *k*_ex_, Δω, and the population in each state can be derived (19).

In our NMR study, we measured *R*_2_ relaxation dispersions of three peptide–HLA-B35*01 complexes, each of which has the same heavy chain and β2M but a different peptide (Figure 2B). In what follows, they are referred to as the high-stable, middle-stable, and low-stable MHC complexes because their melting temperatures are 61.4, 57.5, and 50.1°C, respectively (7). These experiments identified fluctuating residues in the peptide-binding domain of the high-stable MHC complex at 20°C, whereas no fluctuation was observed in the Ig-like domain, which binds to β2M. We fitted all the *R*_2_ relaxation dispersions globally to a two-state exchange model (major ↔ minor), yielding an exchange rate (*k*_ex_ = *k*_major→minor_ + *k*_minor→major_) of 766 ± 41 s^−1^ and a minor population (*p*_minor_) of 4.37 ± 0.09%. Analysis of the chemical shift differences (Δω) showed large-amplitude conformational changes in the peptide-binding domain compared with the other fluctuating regions. The relaxation dispersion experiments with the middle- and low-stable MHCs showed that they also fluctuate in the peptide-binding domain, as was the case for the high-stable MHC. However, fewer residues fluctuated in them than in the high-stable MHC. Fitting of the *R*_2_ relaxation dispersion curves yielded *p*_minor_ of 1.25 ± 0.05% and *k*_ex_ of 742 ± 48 s^−1^ for the middle-stable MHC and *p*_minor_ of 0.58 ± 0.02% and *k*_ex_ of 1,590 ± 96 s^−1^ for the low-stable MHC. The minor populations of both middle- and low-stable MHCs were smaller than the high-stable MHC. Almost all of their Δω values were larger than the corresponding Δω values of the high-stable MHC, suggesting that the conformational changes from the major to the minor states are larger for the middle- and low-stable MHCs than for the high-stable MHC. Furthermore, in order to reveal the role of the fluctuation observed by *R*_2_ relaxation dispersion, we conducted an analysis of the change in the heat capacity (Δ*C*_p_) using *k*_ex_ values obtained from relaxation dispersion experiments at different temperatures. The observed Δ*C*_p_ suggested that dehydration occurs in the minor state due to interaction and/or induced fit of the peptide to the MHC-binding domain. The dehydration in the hydrophobic peptide-binding domain, composed of many fluctuating hydrophobic residues, should contribute to a strong interaction. It is likely that the interaction between the peptide-binding domain and the peptide itself becomes stronger in the more compacted dehydrated minor state. From the above results, we conclude that the minor state is important for the stabilization of the MHC molecule to prevent disintegration of the complex. The rate constants of the major-to-minor transition observed in this study were much faster than the disintegration rate (10), suggesting that the MHC molecule rapidly forms the more compact minor state before it has time to disintegrate.

In nature, a number of different antigenic peptides are loaded onto MHC molecules. Our results suggest that the major state of an MHC molecule would accommodate antigenic peptides loosely relative to the compact minor state, thus allowing different peptides to bind initially. We propose a two-step “transient induced-fit model” for the binding of a peptide to an MHC molecule. In the first step, a peptide is loosely loaded into the binding domain in the major state, and in the second step, conformational tuning to the compact minor state enables the MHC molecule to avoid rapid disintegration (Figure 2C).

## Simulation of the Molecular Dynamics (MD) of MHC Molecules

Molecular dynamics is a useful tool to predict and visualize conformational dynamics at atomic resolution. MD simulation has greatly contributed to our knowledge of the plastic nature of MHC molecules, especially in the free state (20–23), in particular, because the peptide-free form of the MHC moiety is difficult to investigate experimentally. MD studies indicate that the peptide-free form is more flexible than the bound form. Bailey et al. showed that the bound form of the MHC molecule is stabilized either by conformational selection or by induced fit of the peptide, depending on the peptide sequence. Fisette et al. simulated the peptide-free form complexed with a peptide-loading chaperone, which mimics the actual peptide-loading step. This indicated that the MHC molecule undergoes an open–close transition depending on the affinity of the peptide, in the presence of the peptide-loading chaperone. The existence of the open–close transition is consistent with our NMR results. In the context of the conformational dynamics of the peptide-bound form, conformational ensembles of the MHC molecules together with different peptides by means of MD simulations have been elucidated (20).

## Perspectives

Thus far, we have focused on the conformational dynamics of the MHC molecule in the absence of a TCR. Although MHC molecules themselves are important research subjects, due to their function in antigen presentation, it is also crucial to consider their interaction with the TCR. MD simulation studies are ongoing to model the interaction between the MHC and the TCRs. These suggest a scanning mode of action of the TCR across MHC molecules (24). Hawse et al. have argued against using NMR to investigate TCR–MHC interactions in solution (14). They suggested that the motions of the peptide-bound MHC and of the TCR are closely matched. This cooperative motion is supposedly mediated by the MHC molecule that holds the peptide, but little is known in this respect. Because we showed that NMR is suitable for elucidating the conformational dynamics of MHC molecules, we anticipate that further NMR studies on the MHC–TCR complex will provide some insight into this issue as well.

## Author Contributions

All authors listed have made substantial, direct, and intellectual contribution to the work and approved it for publication.

## Conflict of Interest Statement

The authors declare that the research was conducted in the absence of any commercial or financial relationships that could be construed as a potential conflict of interest.
